# The impact of direct-to-consumer wearables in pediatric electrophysiology telehealth clinics: A real-world case series

**DOI:** 10.1016/j.cvdhj.2020.09.005

**Published:** 2020-10-07

**Authors:** Lisa Roelle, Aarti S. Dalal, Nathan Miller, William B. Orr, George Van Hare, Jennifer N. Avari Silva

**Affiliations:** ∗ Division of Pediatric Cardiology, Washington University School of Medicine, St Louis, Missouri; † Cardiac Catheterization Laboratory, St Louis Children’s Hospital, St Louis, Missouri; ‡ Department of Biomedical Engineering, Washington University McKelvey School of Engineering, St Louis, Missouri

**Keywords:** Telehealth, Pediatric electrophysiology, Cardiac wearables, EMR integration

**Key Findings**

• Direct-to-consumer wearable devices can have a positive impact in pediatric electrophysiology telehealth clinics by providing real-time data on heart rate and rhythm in this population. These data can be used to guide medical decision-making on titration of drugs or decision to proceed to ablation therapy.
• Developing simple workflows to securely transmit and integrate these electrocardiographic tracings into the electronic medical record (in these cases, use of the MyChart application and the EPIC electronic medical record) have been described.
• There are important economic, reimbursement, workflow, and data integrity considerations that should be more thoroughly explored in future studies.

## Introduction

Given the current SARS-CoV-2 pandemic, there has been a significant growth of telemedicine programs across all specialties.^1^^,^^2^ Pediatric cardiology, specifically electrophysiology, where the patient population includes pediatric patients with heart rhythm abnormalities, seems uniquely suited to this opportunity. With the increase of commercially available cardiac wearable devices accessible to consumers without medical prescription, the “direct-to-consumer” (D2C) devices, telemedicine visits can be enhanced using real-time cardiac data/diagnostics to supplement diagnosis and management of cardiac arrhythmias. Two prominent D2C devices that provide real-time electrocardiogram (ECG) data include the KardiaMobile (AliveCor, Mountain View, CA) and the Apple Watch Series 5 (Apple, Cupertino, CA). In this report, we present 2 pediatric cases using these D2C cardiac devices and discuss how they have positively impacted the telemedicine and patient experience. The research reported in this paper adheres to the CARE case report guidelines.

## Case report

### Case #1

P.P. is a 20-year-old African-American man who was diagnosed at age 15 with paroxysmal atrial fibrillation (AF) after presenting to the emergency department with chest pain during exertion. He had an extensive workup including a normal echocardiogram and a diagnostic electrophysiology study (EPS), which demonstrated no inducible arrhythmias. Over the next several yearly office visits, he reported having clinical episodes of AF approximately once a year, lasting seconds to minutes, that always spontaneously converted back to normal sinus rhythm. There had been no documentation of these episodes.

**Figure 1:**
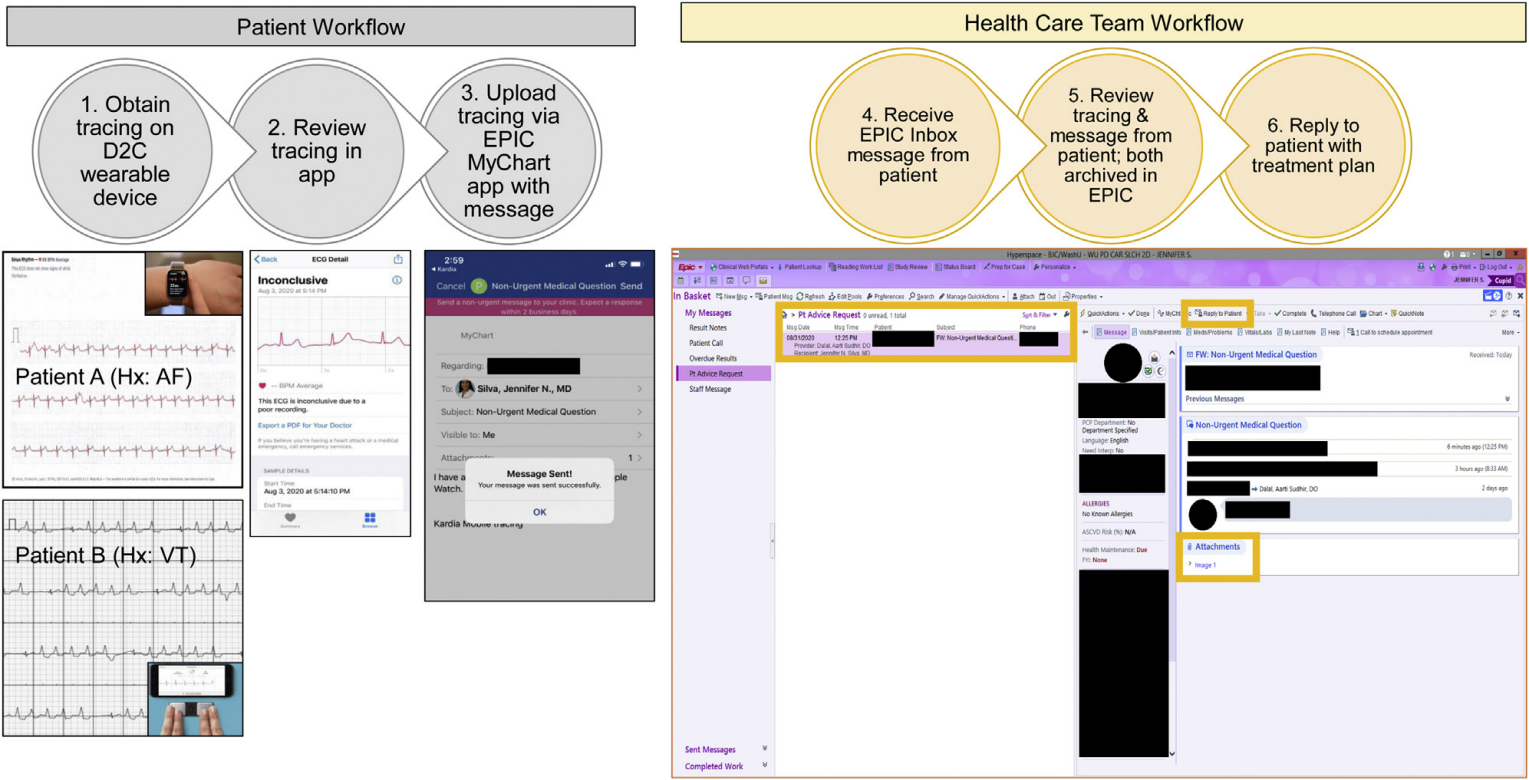
Patient and physician workflow for obtaining and transmitting electrocardiogram (ECG) tracings from direct-to-consumer (D2C) wearable devices. The gray workflow, or “Patient Workflow,” clarifies the stepwise approach the patient takes to obtain the ECG tracing, review the tracing, and upload the tracing via the EPIC MyChart application. The screen shot images associated with each step demonstrate what the patient sees at each step. The orange workflow, or “Health Care Team Workflow,” delineates how the message appears in the EPIC inbox (Patient Advice Request>>Non-Urgent Medical Question), where the attachment hyperlink is shown, and how to reply to the patient. AF = atrial fibrillation; VT = ventricular tachycardia.

This year, P.P. had a telehealth visit with his pediatric cardiac electrophysiology team. During that visit, he reported having episodes of “skipped beats” that lasted for only a few seconds, but no concerns for AF. He did report that he recently had purchased a new Apple Watch Series 5 but had not set up the ECG application in the Health App. During his visit, the team assisted the patient in setting up the application and encouraged him to obtain a real-time, “on-demand” ECG tracing using his Apple Watch (Figure 1). The Apple Watch Series 5 has the ability to provide continuous heart rate trends in addition to obtaining a single-lead ECG, or “on-demand” recording. This tracing was then e-mailed to the team, by whom a real-time assessment of his heart rate and rhythm could be performed, confirming that he was in normal sinus rhythm with a rate of 89 beats per minute (bpm). In addition, the team worked with the patient to establish a workflow for submission and evaluation of future tracings using the electronic medical record, in this case using the EPIC MyChart functionality, whereby he could securely send tracings and questions to his health care team and the team could reply using a HIPAA-compliant mode of communication when the patient had symptoms concerning for AF.

### Case #2

P.G. is a 14-year-old female patient with a past medical history of hypothyroidism who presented to the emergency department with an irregular heartbeat, after having tracked her heart rate by pulse assessment and pulse oximeter, between 80 and 140 bpm and associated intermittent chest pain with palpitations. Initial ECG revealed wide complex tachycardia with left bundle branch block and inferior axis, consistent with ventricular tachycardia (VT) arising from the right ventricular outflow tract (RVOT). She underwent EPS and transcatheter ablation and had an acutely successful radiofrequency ablation of her RVOT-VT procedure with recurrence of her clinical VT several hours post procedure. The patient was started on oral verapamil and titrated to a therapeutic dose of 120 mg every 8 hours, with significant decreased premature ventricular contraction (PVC) burden and VT. The family purchased an AliveCor KardiaMobile for assessment of her heart rate and rhythm at home during symptomatic events. Teaching was provided prior to discharge for family to understand the differences between sinus rhythm tracings and VT on the KardiaMobile, as well as workflow teaching for transmission of tracings using MyChart to allow for a secure, HIPAA-compliant transmission.

After hospital discharge, the patient/parent transmitted multiple ECG tracings from the KardiaMobile demonstrating normal sinus rhythm, PVCs, and nonsustained VT (Figure 1), which has helped guide her outpatient medical management and drug titration. After consecutive tracings demonstrated nonsustained VT, the decision was made to pursue repeat EPS. Following repeat ablation of her RVOT-VT, follow-up KardiaMobile tracings demonstrated normal sinus rhythm with sinus arrhythmia with no PVCs at rates of 62 to 74 bpm. Symptomatic tracings recorded for concerns of chest pain or skipped beats confirmed sinus rhythm with no PVC recurrence. The most recent transmission acquired at the start of a telehealth visit demonstrated sinus rhythm and guided further conversations around ongoing arrhythmia concerns and management.

## Discussion

In this report, we describe 2 cases in which D2C cardiac wearable ECG devices, the Apple Watch Series 5 and the KardiaMobile, were used during telehealth visits to provide real-time assessment of the patient’s heart rate and rhythm. These cases represent the powerful potential role these products may have to support and improve pediatric cardiac electrophysiology telehealth visits. Integrating new, commercially available technologies into electrophysiology telemedicine clinics will be an important value-add to deriving the most benefit from these types of visits by improving physician-patient communication, diagnosis, and treatment of cardiac arrhythmias. Pediatric patients may be ideally suited to these visits and technologies, as they are often technologically savvy. Additionally, pediatric patients may have access to their parent’s D2C wearable device for obtaining ECG tracings.

Patient access to these technologies represents a significant barrier to adoption. As these are D2C marketed devices, it is not necessary for the physician to prescribe the device. Cardiologists can play a critical role in educating patients on available technologies, advantages and disadvantages of each, and how certain technologies may be better for specific patients based on their individual clinical situation. For instance, in those patients where rhythm discrimination is paramount, ECG monitors are preferable; in those patients where rate determination is the primary driver for clinical decision-making, photoplethysmography devices may be better applicable.

Economic barriers may also represent a financial hurdle to widespread adoption and implementation of these devices. The Kardia single-lead ECG monitor is currently available for $89 and is eligible for reimbursement through a flexible spending account (FSA) or health savings account (HSA). More recently, a 6-lead Kardia ECG monitor has received FDA approval and is available to consumers for $149, also eligible for FSA/HSA reimbursement. In contrast, the Apple Watch Series 5 is currently available for $399 and is not eligible for FSA/HSA reimbursement. For patients that depend on insurance or FSA reimbursement to cover medical costs, the KardiaMobile may represent a more economical option. Both systems require a paired smartphone for full functionality, which impacts the total cost. Patients and families with more disposable income may be more likely to invest in a product that will provide insight into their symptoms, diagnosis, and treatment regardless of insurance reimbursement. Additionally, for some patients, there may be personal stylistic preferences that may drive decision-making about cardiac wearables.

Simple workflows for secure transmission of data and tracings should be created and provided to patients. Our proposed solution has been to encourage patients to obtain an ECG tracing on the day of and prior to their telehealth visit, and to utilize the MyChart application, embedded within our electronic medical record, EPIC, to upload the data for review prior to/during their telehealth visit. Both the KardiaMobile and Apple Watch Series 5 allow the user to save an ECG strip and easily upload tracings into the MyChart application via direct messaging to the health care team for provider review (Figure 1).

Previous research has shown that the KardiaMobile provides reliable data to assist in the diagnosis of pediatric cardiac arrhythmias.^3^^,^^4^ In contrasting, there is little data on the reliability of ECG data from ECGs obtained from the Apple Watch Series 5. A comparative study assessing diagnostic quality of the Apple Watch Series 5 ECG compared to standard 12-lead ECGs would provide important data to support its use in an electrophysiology telehealth clinic.

## Conclusion

These cases demonstrate the critical adjunctive role D2C cardiac wearable devices may have in telemedicine. Although there are economic, reimbursement, workflow, and data integrity considerations that have yet to be thoroughly explored and defined, these tools will impact the implementation of telehealth, particularly in pediatric electrophysiology telehealth clinics. Long-term patient compliance with these devices should be assessed in future studies.

## Funding Sources

The authors have no funding sources to disclose.

## Disclosures

The authors have no relevant disclosures for the above case report. In the spirit of full disclosure, J.N.A.S. has previously received grant support (financial and device-based support) from AliveCor, Inc, that is not related to the current cases presented.
